# Examining relationships between perceived neighborhood social cohesion and ideal cardiovascular health and whether psychosocial stressors modify observed relationships among JHS, MESA, and MASALA participants

**DOI:** 10.1186/s12889-022-14270-x

**Published:** 2022-10-11

**Authors:** Akilah J. Dulin, Jee Won Park, Matthew M. Scarpaci, Laura A. Dionne, Mario Sims, Belinda L. Needham, Joseph L. Fava, Charles B. Eaton, Alka M. Kanaya, Namratha R. Kandula, Eric B. Loucks, Chanelle J. Howe

**Affiliations:** 1 Center for Health Promotion and Health Equity, Brown University, Providence, RI USA; 2 Center for Epidemiologic Research, Department of Epidemiology, Brown University, Providence, RI USA; 3 Hassenfeld Child Health Innovation Institute, Brown University, Providence, Rhode Island USA; 4 Department of Social Medicine, Population and Public Health, University of California Riverside School of Medicine, Riverside, CA USA; 5 Department of Epidemiology, University of Michigan, Ann Arbor, MI USA; 6 Centers for Behavioral and Preventive Medicine, The Miriam Hospital, Providence, RI USA; 7 Department of Family Medicine, Warren Alpert Medical School of Brown University, Providence, RI USA; 8 Center for Primary Care and Prevention Kent Memorial Hospital, Warwick, RI USA; 9 Division of General Internal Medicine, University of California, San Francisco, San Francisco, CA USA; 10 Department of Internal Medicine, Northwestern University, Chicago, IL USA; 11 Center for Health Promotion and Health Equity Research, Brown University School of Public Health, Box G-S121-8, 02912 Providence, RI USA

**Keywords:** Neighborhood, Resilience, Psychosocial factors, Life’s simple 7, Cardiovascular health

## Abstract

**Background:**

Psychosocial stressors increase the risks for cardiovascular disease across diverse populations. However, neighborhood level resilience resources may protect against poor cardiovascular health (CVH). This study used data from three CVH cohorts to examine longitudinally the associations of a resilience resource, perceived neighborhood social cohesion (hereafter referred to as neighborhood social cohesion), with the American Heart Association’s Life’s Simple 7 (LS7), and whether psychosocial stressors modify observed relationships.

**Methods:**

We examined neighborhood social cohesion (measured in tertiles) and LS7 in the Jackson Heart Study, Multi-Ethnic Study of Atherosclerosis, and Mediators of Atherosclerosis in South Asians Living in America study. We used repeated-measures, modified Poisson regression models to estimate the relationship between neighborhood social cohesion and LS7 (primary analysis, n = 6,086) and four biological metrics (body mass index, blood pressure, cholesterol, blood glucose; secondary analysis, n = 7,291). We assessed effect measure modification by each psychosocial stressor (e.g., low educational attainment, discrimination).

**Results:**

In primary analyses, adjusted prevalence ratios (aPR) and 95% confidence intervals (CIs) for ideal/intermediate versus poor CVH among high or medium (versus low) neighborhood social cohesion were 1.01 (0.97–1.05) and 1.02 (0.98–1.06), respectively. The psychosocial stressors, low education and discrimination, functioned as effect modifiers. Secondary analyses showed similar findings. Also, in the secondary analyses, there was evidence for effect modification by income.

**Conclusion:**

We did not find much support for an association between neighborhood social cohesion and LS7, but did find evidence of effect modification. Some of the effect modification results operated in unexpected directions. Future studies should examine neighborhood social cohesion more comprehensively and assess for effect modification by psychosocial stressors.

**Supplementary Information:**

The online version contains supplementary material available at 10.1186/s12889-022-14270-x.

## Background

Cardiovascular disease (CVD) is the leading cause of death globally and it is well-established that behavioral and physiological health factors and psychosocial stressors increase the risks for CVD across diverse populations (e.g., racially, ethnically, gender, geographically) [1–3]. The American Heart Association’s Life’s Simple 7 (LS7) measure describes behavioral and physiological indicators including smoking, diet quality, physical activity, body mass index, fasting glucose, blood pressure and total cholesterol as modifiable CVD risk factors that should be monitored and addressed [4]. Along with screening for LS7, professional societies recommend that patients receive screening and counseling for psychosocial stressors [5]. Some of these psychosocial stressors include global stress, discrimination, anger, hostility and depression [6]. In addition to these psychosocial stressors, features of the neighborhood environment impact cardiovascular health (CVH). Specifically, low neighborhood socioeconomic status (hereafter referred to as neighborhood deprivation) functions in part as a proxy for neighborhood psychosocial stress that adversely impacts LS7 metrics and incident CVD.

There are several direct and indirect pathways through which psychosocial stressors increase CVD risk. For instance, exposure to acute and chronic psychosocial stressors may lead to poor coping responses such as smoking, poor diet quality and physical inactivity [6]. In turn, these poor health behaviors are related to hypertension, elevated blood glucose, total cholesterol and higher BMI [6]. Another mechanism occurs via allostatic load. Allostatic load refers to dysregulation of physiological systems such as the hypothalamic pituitary adrenal axis and metabolic systems-thyroid axis, for example, that help maintain the body’s stability during stress [7]. Chronic psychosocial stressor exposure may lead to prolonged and repeated activation of these systems which results in chronic overactivity or underactivity that increases CVD risk (e.g., via increased inflammatory cytokines, hypertension, diabetes) [8]. Also, psychosocial stressors may increase CVD incidence directly. Prior findings indicate that severe, sudden acute stressful events such as natural disasters or major emotional events result in short-term, marked, increases in CVD events [9]. However, despite the substantial effects of psychosocial stressors on CVD risk, protective factors (e.g., resilience resources) may reduce the risks for poor CVH outcomes [2].

Resilience resources may protect against poor CVH outcomes via engagement in more positive health behaviors and reduction in the risks for poorer physiological functioning [10, 12]. Resilience refers to the ability to adapt positively despite acute and chronic adversities whereby a person draws upon resources from a variety of sources (e.g., individual, interpersonal and structural) to overcome these adversities [11]. In a recent systematic review and meta-analysis, individual and interpersonal-level resilience resources such as optimism and social support are associated favorably with CVH [10]. This review also noted that despite advancements in this important area of research, gaps remain.

Specifically, some of the gaps in resilience research relate to limited generalizability, analytical approach, time frame and the level of resilience resource examined. Much of the research is conducted in populations with diagnosed CVD or does not include racially, ethnically, socioeconomically or geographically diverse populations [10]. Additionally, resilience is grounded in experiencing adversities (e.g., psychosocial stressors) and drawing upon a resilience resource(s) to overcome these adversities. However, little research examines multilevel psychosocial stressors and resilience resources together in analytic models. Further, the preponderance of evidence is based on cross-sectional studies. Also, the majority of research focuses on individual- and interpersonal-level resilience resources rather than neighborhood-level resources [10].

Although some research examines neighborhood-level resilience (e.g., social cohesion), findings are mixed. Neighborhood social cohesion is a multicomponent concept that includes the extent to which neighborhoods [1] promote inclusion and reduce social inequities and disparities (e.g., income/wealth, race/ethnicity) and [2] build or strengthen social capital [13, 14]. Specifically, neighborhood social cohesion measures typically examine the extent to which neighborhood residents interact with each other and the attributes that arise such as shared attitudes and norms, reciprocity, trust, sense of belonging, cooperation, social support and collective action to address stressors that impact the community [15–17]. These measures capture perceived (e.g., self-report measures examined solely at the individual level or individual-level responses aggregated to the neighborhood level) or objective (e.g., administrative measures of income equality) neighborhood social cohesion [13, 16, 18]. It is expected that neighborhood social cohesion promotes health via increased spread of health information, collective social norms for healthy behaviors, collective action to advocate for health promoting resources and social support during times of stress [19]. While there is support for neighborhood social cohesion on individual CVH measures, less is known about its role on overall CVH profiles, like LS7 [18]. Additionally, it is unclear how neighborhood social cohesion may impact CVH over time. To address the above-mentioned research gaps, we propose two specific study objectives. The first objective is to use harmonized data from three diverse CVH cohort studies to examine whether perceived neighborhood social cohesion (hereafter referred to as neighborhood social cohesion) is associated with LS7 and the 4 LS7 biological metrics as a combined measure over time. The second objective is to use the harmonized data to identify whether psychosocial stressors (e.g., anger, discrimination, low neighborhood safety) are effect modifiers of the observed relationships between neighborhood social cohesion and LS7 outcomes.

## Methods

### Study population

We used a harmonized dataset from three cohort studies in the United States – Jackson Heart Study (JHS), Multi-Ethnic Study of Atherosclerosis (MESA), and Mediators of Atherosclerosis in South Asians Living in America (MASALA). JHS is a study with 5,306 African American adults over the age of 21 and consists of three exams approximately every 4 years in addition to annual follow-up interviews every 12 months. MESA is a study among 6,814 racially/ethnically diverse adults over the age of 45 without a history of CVD at study enrollment. We used data from the first five exams which span 10 years. MASALA is a study of 906 South Asian adults over the age of 40 without CVD history at study enrollment. MASALA consists of two exams. Detailed description of the cohort study designs are detailed elsewhere [20–22]. The Institutional Review Boards (IRBs) at each site approved the cohort studies and all study participants provided written informed consent. Additionally, the Brown University IRB approved our secondary analysis study.

### Measures

The exposure is based on participants’ reports of neighborhood social cohesion at MESA/MASALA Exam 1 and JHS Third Annual Follow-up Interview (Cronbach’s alpha = 0.73 in the harmonized dataset). This is a five-item measure assessing close-knit neighborhood, willingness to help, getting along, trust, and sharing the same values [15]. We created tertiles for analyses. For JHS, the interview took place three years after Exam 1 based on the JHS design.

The primary outcome is a binary variable of ideal and intermediate versus poor CVH defined by the composite LS7 metrics. The LS7 is based on self/proxy-reported and/or physical exams. A score of 0, 1, and 2 for poor, intermediate, and ideal, respectively, is assigned for each metric and summed across all of them (range: 0–14). Scores of 8–14 indicate ideal or intermediate CVH. Details on each LS7 metric are described elsewhere [23]. For the primary outcome, we used data from MESA Exams 1 and 5 and MASALA Exams 1 and 2; JHS did not collect all LS7 metrics concurrent with or after exposures assessment. We conducted secondary analyses using a subset of LS7 metrics available in all three cohorts, MESA, JHS and MASALA. The secondary outcomes used repeated measures of four biological LS7 metrics (BMI, blood pressure, glucose, and cholesterol) assessed at all exams concurrent with or after exposure assessment. We examined two secondary outcome variables: (1) Ideal/intermediate metrics versus at least one poor metric, and (2) 0–1 poor metrics versus 2–4 poor metrics [24, 25].

Potential confounding variables include age, sex/gender, race, ethnicity, geographical region, nativity, marital status, self-rated health, health insurance type, self- and family-history of CVD and social support [26, 27]. All confounders were self-reported measured at Exam 1 and considered a source of potential selection and confounding bias.

Potential effect modifiers include the following psychosocial stressors - self-reported anger [28], depressive symptoms (yes/no) [29], chronic stress [30], education (less than high school/high school or some college/college degree or higher), employment status (unemployed/employed at least part-time), income ($0-$19,999/$20,000-$49,999/$50,000+), discrimination [31], neighborhood safety (safe/not safe) and US Census derived indicators of neighborhood deprivation [32]. All effect modifiers were assessed at Exam 1 and examined as tertiles (low/medium/high – unless otherwise stated).

Supplemental Fig. 1 shows the relationship between all variables included in the study in a causal directed acyclic graph. We selected variables as covariates if they were considered to be sources of confounding or selection bias or potential effect modifiers based on prior literature [18, 33–35].

### Statistical analyses

We excluded participants who did not have available data for neighborhood social cohesion, potential confounders or sources of selection bias, potential effect modifiers, and outcomes at relevant time points. We used Chi-squared and Wilcoxon Mann-Whitney tests to compare Exam 1 characteristics between the included and excluded participants. Because the follow-up time between exams differed within and across cohorts, we constructed two equal bins of follow-up time that corresponded to 6-year intervals. If participants had multiple outcome assessments within an interval, we used the furthest observation in analysis. Outcomes assessed concurrently with the exposure were included. Hereafter, ‘visit’ refers to a given 6-year interval and ‘exam’ refers to the cohort exams. We treated death (9.3% and 7.7% in the primary and secondary analysis sample, respectively) as a censoring event and not an event that created undefined CVH outcomes [36, 37].

In primary analyses, we used repeated-measures, modified Poisson regression models to examine the overall relationship between neighborhood social cohesion and CVH. Unadjusted and adjusted models included neighborhood social cohesion, visit, and product terms between neighborhood social cohesion and visit as independent variables. Also, we included potential confounders and effect modifiers in the adjusted models to account for confounding and selection bias. The abovementioned primary analyses were repeated excluding the neighborhood social cohesion and visit product terms. To assess for effect measure modification by psychosocial stressors, we modified the adjusted models to include a product term between neighborhood social cohesion, visit and the psychosocial stressor of interest. When we assessed for effect measure modification by one psychosocial stressor measure at a time, we included the other psychosocial stressor measures in the outcome models to adjust for confounding and selection bias. Global chi-squared tests indicated whether at least one of the relevant product term coefficients was different from zero. Secondary analyses repeated the primary analyses but assessed for the two secondary CVH outcomes separately.

In our primary and secondary analyses, the continuous variable (age) was fitted using restricted quadratic splines with 4 knots at unequal intervals (i.e., 5th, 35th, 65th, 95th percentiles) [38]. All modified Poisson regression models accounted for within neighborhood clustering of data (i.e., census tract at Exam 1) and we specified independent working correlation structures. For sensitivity analysis, we repeated all primary and secondary analyses solely accounting for observations correlated within individuals and specified exchangeable working correlation structures. Last, we examined whether the overall relationship differed by cohort.

Interpretation of our findings aligns with recent literature on significance and hypothesis testing [39, 40]. Specifically, we base our interpretations on data compatibility using point estimates, confidence intervals, and p-values, rather than relying solely on statistical significance. All statistical analyses were carried out using SAS 9.4 (SAS Institute, Inc., Cary, NC).

## Results

### Primary analysis

The primary analysis included 6,086 participants (**Supplemental Fig. 2**). Table 1 highlights the characteristics among the included and excluded participants. The included participants had lower perceived neighborhood social cohesion than excluded participants. Specifically, for the included participants, 25.5% were in the high neighborhood social cohesion group, 40.3% in medium and 34.1% in low. The median (25th percentile-75th percentile) age was higher among included [61 (53–69)] than excluded participants [53 (44–62)]. Most included participants were female (51.7%), White, non-Hispanic (37.6%), born in the US (64.8%), and lived in the Midwest (38.2%). Compared to excluded participants, included participants were more likely to be married (64.2% versus 59.6%), report good health (91.1% versus 75.9%), have public or private health insurance (91.6% versus 87.6%) but less likely to be employed (50.7% versus 61.5%) and have a high school education or some college degree (44.0% versus 46.1%). Income levels were similar between the included and excluded participants. Further, the included participants, compared to the excluded, reported fewer depressive symptoms (87.8% versus 79.2%), included participants also were more likely to report that their neighborhood was safe (85.3% versus 59.9%).

**Table 1:** Characteristics of MASALA and MESA participants at the exam concurrent with neighborhood social cohesion assessment

Characteristics	Included (n = 6,086)		Excluded (n = 1,661)		P-value^*^
	N	%	N	%	
**Neighborhood social cohesion** ^†^ **at Exam 1**					
Low	2,078	34.1	382	23.0	< 0.01
Medium	2,455	40.3	502	30.2	
High	1,553	25.5	777	46.8	
**Age** ^‡^ **in years at Exam 1**	61 (53–69)		53 (44–62)		< 0.01
**Sex/gender at Exam 1**					
Female	3,145	51.7	1,066	64.2	< 0.01
Male	2,941	48.3	595	35.8	
**Race/ethnicity at Exam 1**					
White non-Hispanic	2,288	37.6	0	0	< 0.01
Asian	1,081	17.8	15	0.9	
African American	1,460	24.0	1,646	99.1	
Hispanic	1,257	20.7	0	0	
**Nativity at Exam 1**					
Other	2,145	35.2	15	0.9	< 0.01
U.S.-born	3,941	64.8	1,646	99.1	
**Region at Exam 1**					
West	1,126	18.5	0	0	< 0.01
South	870	14.3	1,646	99.1	
Midwest	2,325	38.2	15	0.9	
Northeast	1,765	29.0	0	0	
**Marital Status at Exam 1**					
Never married, separated/divorced, widowed	2,178	35.8	671	40.4	< 0.01
Married	3,908	64.2	990	59.6	
**Self-rated health** ^§^ **at Exam 1**					
Not good	542	8.9	401	24.1	< 0.01
Good	5,544	91.1	1,260	75.9	
**Health Insurance at Exam 1**					
None	509	8.4	206	12.4	< 0.01
Public or Private	5,577	91.6	1,455	87.6	
**Family history of CVD and stroke at Exam 1**					
No	2,676	44.0	692	41.7	0.09
Yes	3,410	56.0	969	58.3	
**Education at Exam 1**					
Less than high school	949	15.6	156	9.4	< 0.01
High school or some college	2,675	44.0	766	46.1	
College degree or more	2,462	40.5	739	44.5	
**Employment at Exam 1**					
Unemployed	3,002	49.3	639	38.5	< 0.01
Employed (Part/full-time)	3,084	50.7	1,022	61.5	
**Income at Exam 1**					
$0-$19,999	1,299	21.3	364	21.9	0.88
$20,000-$49,999	2,181	35.8	593	35.7	
$50,000+	2,606	42.8	704	42.4	
**Anger** ^†^ **at Exam 1**					
Low	2,410	39.6	499	30.0	< 0.01
Medium	2,053	33.7	433	26.1	
High	1,623	26.7	729	43.9	
**Depressive symptoms (CES-D ≥ 16) at Exam 1**					
No	5,344	87.8	1,315	79.2	< 0.01
Yes	742	12.2	346	20.8	
**Chronic stress** ^†^ **at Exam 1**					
Low	3,064	50.4	280	16.9	< 0.01
Medium	1,960	32.2	650	39.1	
High	1,062	17.5	731	44.0	
**Discrimination** ^†^ **at Exam 1**					
Low	2,431	39.9	365	22.0	< 0.01
Medium	2,076	34.1	500	30.1	
High	1,579	25.9	796	47.9	
**Neighborhood deprivation** ^†^ **at Exam 1**					
Low	1,471	24.2	822	49.5	< 0.01
Medium	2,186	35.9	495	29.8	
High	2,429	39.9	344	20.7	
**Neighborhood safety at Exam 1**					
Safe	5,193	85.3	995	59.9	< 0.01
Not safe	893	14.7	666	40.1	
**Social support at Exam 1**					
Not high	2,322	38.2	425	25.6	< 0.01
High	3,764	61.9	1,236	74.4	

^*^ Pearson’s χ^2^-test and Wilcoxon-Mann-Whitney test

^†^ Tertiles are not 33% due to ties at boundaries and participants with the same value were included in the same category

^‡^ Median **(**25th percentile-75th percentile)

^**§**^ Binary variable for self-rated health was used to indicate ‘Good’ and ‘Not good’ categories due to the harmonization of different self-rated health measures across JHS, MESA, and MASALA cohorts

Abbreviations: CVD, cardiovascular disease; CVH, cardiovascular health; MASALA, Mediators of Atherosclerosis in South Asians Living in America; MESA, Multi-Ethnic Study of Atherosclerosis

Table 2 shows adjusted prevalence ratios (aPR) for ideal/intermediate versus poor CVH. Focusing on the overall findings across visits that were most compatible with the data, high and medium (versus low) neighborhood social cohesion was not associated with ideal/intermediate CVH (aPR: 1.01, 95% CI: 0.97–1.05, and aPR: 1.02, 95% CI: 0.98–1.06). The corresponding 95% CIs indicate that a weak positive association and a weak negative association was compatible with the data. Results by visit yielded similar findings. There was some evidence for effect measure modification by education and discrimination levels (Table 3**)**. For instance, high (versus low) neighborhood social cohesion was negatively associated with ideal/intermediate (versus poor) CVH among participants with less than high school education (aPR: 0.77, 95% CI: 0.67–0.88). However, a positive association was most compatible with the data among those who had high school or some college (aPR: 1.05, 95% CI: 0.98–1.12) or college degree or more (aPR: 1.05, 95% CI: 1.00-1.10). Findings by visit were similar (**Supplemental Table 6**).

**Table 2:** Prevalence ratios (PR) for CVH outcomes^*^ during exams concurrent or subsequent to neighborhood social cohesion

Outcome	Neighborhood social cohesion and visit product term in outcome model		High versus low neighborhood social cohesion PR (95% CI)		Medium versus low neighborhood social cohesion PR (95% CI)	
			**Unadjusted**	**Adjusted** ^†^	**Unadjusted**	**Adjusted** ^†^
**Primary (n = 6,086)**						
Ideal or intermediate versus poor CVH	Without product term		1.06 (1.01–1.10)	1.01 (0.97–1.05)	1.07 (1.03–1.11)	1.02 (0.98–1.06)
	With product term^‡^	Visit 1	1.06 (1.01–1.10)	1.01 (0.96–1.05)	1.08 (1.01–1.12)	1.02 (0.98–1.06)
		Visit 2	1.06 (0.99–1.12)	1.02 (0.96–1.08)	1.06 (1.03–1.12)	1.02 (0.96–1.07)
**Secondary (n = 7,291)**						
Ideal or intermediate (but no poor) metrics versus 1 or more poor metrics	Without product term		1.00 (0.93–1.08)	1.01 (0.95–1.09)	1.06 (1.00-1.13)	1.01 (0.95–1.07)
	With product term^§^	Visit 1	0.99 (0.92–1.07)	1.02 (0.95–1.09)	1.04 (0.98–1.11)	0.99 (0.93–1.05)
		Visit 2	1.03 (0.94–1.12)	1.00 (0.92–1.10)	1.09 (1.01–1.19)	1.03 (0.95–1.12)
Lower cardiovascular risk (0–1 poor metrics) versus non-lower cardiovascular risk (2–4 poor metrics)	Without product term		0.99 (0.97–1.02)	0.99 (0.97–1.02)	1.03 (1.00-1.05)	1.01 (0.98–1.03)
	With product term^¶^	Visit 1	0.99 (0.96–1.02)	0.99 (0.96–1.03)	1.03 (1.01–1.06)	1.01 (0.99–1.04)
		Visit 2	1.01 (0.97–1.04)	0.99 (0.96–1.03)	1.01 (0.98–1.04)	0.99 (0.96–1.02)

Note: Each modified Poisson regression model accounted for clustering within neighborhood (i.e., census tract at Exam 1) [47]

^*^ Primary CVH outcomes were measured during Exams 1 and 2 in MASALA and Exams 1 and 5 in MESA. Secondary CVH outcomes included all cohort exams in JHS, MASALA, and MESA, except for JHS Exam 1 because exposure was assessed after JHS Exam 1

^†^ Each outcome model using modified Poisson regression was adjusted for age, sex/gender, race, nativity, geographic region, marital status, self-rated health, insurance, family CVD history, social support, education level, income, employment, anger, depressive symptoms, chronic stress, discrimination, neighborhood deprivation, and neighborhood safety

^‡^ Neighborhood social cohesion and visit product term coefficients for unadjusted model: -0.01, 0.002, p = 0.82; adjusted model: -0.005, 0.01, p = 0.76

^§^ Neighborhood social cohesion and visit product term coefficients for unadjusted model: 0.05, 0.03, p = 0.47; adjusted model: 0.05, -0.01, p = 0.32

^¶^ Neighborhood social cohesion and visit product term coefficients for unadjusted model: -0.02, 0.02, p = 0.10; adjusted model: -0.02, -0.002, p = 0.31

**Table 3:** Assessment of effect measure modification of adjusted prevalence ratios^*^ (aPR) for ideal or intermediate versus poor CVH**

Psychosocial risk measure (Potential effect measure modifier)	High versus low neighborhood social cohesion and ideal or intermediate versus poor CVH by level of psychosocial risk measure		Medium versus low neighborhood social cohesion and ideal or intermediate versus poor CVH by level of psychosocial risk measure		p^†^	
	**aPR**	**95% CI**	**aPR**	**95% CI**		
**Education at Exam1**						
College degree or more	1.05	(1.00-1.10)	1.04	(0.99–1.09)	< 0.01	
High school or some college	1.05	(0.98–1.12)	1.03	(0.96–1.09)		
Less than high school	0.77	(0.67–0.88)	0.98	(0.89–1.08)		
**Employment at Exam1**						
Employed	1.02	(0.96–1.08)	1.02	(0.96–1.08)	0.81	
Unemployed	1.00	(0.95–1.06)	1.02	(0.97–1.08)		
**Income at Exam1**						
$50,000+	1.03	(0.98–1.08)	1.01	(0.96–1.07)	0.33	
$20,000-$49,999	0.99	(0.92–1.06)	1.01	(0.95–1.07)		
$0-$19,999	0.98	(0.88–1.09)	1.07	(0.99–1.15)		
**Anger at Exam1**						
Low	1.03	(0.97–1.09)	1.04	(0.98–1.10)	0.51	
Medium	1.01	(0.96–1.07)	0.99	(0.94–1.05)		
High	0.98	(0.90–1.06)	1.03	(0.96–1.11)		
**Depressive symptoms at Exam1**						
No	1.02	(0.97–1.06)	1.02	(0.98–1.06)	0.71	
Yes	0.96	(0.84–1.10)	1.03	(0.93–1.14)		
**Chronic stress at Exam1**						
Low	1.02	(0.96–1.07)	1.04	(0.99–1.08)	0.88	
Medium	1.00	(0.93–1.07)	1.00	(0.93–1.07)		
High	1.03	(0.94–1.13)	1.01	(0.92–1.11)		
**Discrimination at Exam1**						
Low	0.96	(0.91–1.02)	0.99	(0.94–1.04)	0.12	
Medium	1.02	(0.96–1.09)	1.02	(0.96–1.07)		
High	1.08	(1.01–1.15)	1.08	(1.01–1.15)		
**Neighborhood deprivation at Exam1**						
Low	0.99	(0.89–1.09)	1.01	(0.93–1.09)	0.67	
Medium	1.00	(0.93–1.08)	1.04	(0.97–1.11)		
High	1.03	(0.98–1.08)	1.02	(0.97–1.06)		
**Neighborhood safety at Exam1**						
Safe	1.00	(0.96–1.04)	1.01	(0.97–1.06)	0.35	
Not safe	1.09	(0.98–1.21)	1.05	(0.97–1.15)		

Note: Each modified Poisson regression model accounted for clustering within neighborhood (i.e., census tract at Exam 1) [47]

^*^ Adjusted for visit, age, sex/gender, race, nativity, geographic region, marital status, self-rated health, insurance, family CVD history, social support, education, income, employment, anger, depressive symptoms, chronic stress, discrimination, neighborhood deprivation, and neighborhood safety

** CVH assessed during exams at or after neighborhood social cohesion assessment at Exam 1 among MASALA and MESA participants included in the primary analysis (n = 6,086)

^†^ Global chi-squared test provided p-values to indicate whether at least one of the coefficients of the product terms between neighborhood social cohesion and psychosocial risk were different from zero

### Secondary analysis

In total, 7,291 participants were included in the secondary analysis (**Supplemental Fig. 3).** Characteristics of these participants are shown in **Supplemental Tables 1**, and were similar to the primary analysis with slightly different distributions.

The aPRs (95% CIs) for ideal/intermediate (but no poor) metrics among high and medium (versus low) neighborhood social cohesion were 1.01 (0.95–1.09) and 1.01 (0.95–1.07), respectively, suggesting a null relationship based on the most compatible estimate **(**Table 2). Similar findings emerged when examining 0–1 versus 2–4 poor LS7 metrics (aPR: 0.99, 95% CI: 0.97–1.02, and aPR: 1.01, 95% CI: 0.98–1.03, for high and medium neighborhood social cohesion respectively). Findings by visit for both secondary outcomes were similar. When examining ideal/intermediate (but no poor) metrics (**Supplemental Table 2**), there was evidence for effect modification by psychosocial stressors such as lower education, lower income, and greater discrimination. Similarly, when examining 0–1 poor versus 2–4 poor metrics, there was evidence for effect modification by income (**Supplemental Table 3**). Findings by visit were similar (Supplemental Tables 7–8).

### Sensitivity analysis

Again, focusing on the estimates most compatible with the data, inferences based on the primary and secondary analysis did not change when we solely accounted for outcomes correlated within individuals or models were specified with an exchangeable working correlation structure. Cohort-stratified findings for primary analyses showed that the aPRs for high (versus low) neighborhood social cohesion were consistently higher in MASALA compared to MESA based on the most compatible estimates (**Supplemental Table 4**). The aPRs for medium versus low neighborhood social cohesion were similar across cohorts, except for MASALA at visit 2. Cohort-stratified findings for the secondary analysis examining ideal/intermediate (but no poor metrics) showed similar aPRs for JHS and MASALA, while MESA had consistently null findings for high versus low neighborhood social cohesion. When comparing medium versus low cohesion, aPRs differed across all cohorts. For 0–1 poor metrics versus 2–4 poor metrics, aPRs for JHS were consistently lower than those in MESA and MASALA (**Supplemental Table 5**).

## Discussion

This study addresses some of the gaps in the literature by examining the role of a neighborhood-level resilience resource, perceived neighborhood social cohesion, on CVH outcomes over time and in the presence of adversities (i.e., psychosocial risks) among racially, ethnically and geographically diverse participants. We hypothesized that perceived neighborhood social cohesion (e.g., via interactions with neighborhood residents, social support, collective action, shared norms) would be a resilience resource that results in better CVH. However, analyses revealed little support for relationships between neighborhood social cohesion and LS7. In contrast, there was support for effect modification by some of the psychosocial stressors. We expected that individuals experiencing more adversity would benefit more from mobilizing resilience resources, but this direction was not supported across all psychosocial stressors that emerged as effect modifiers. For instance, higher levels of neighborhood social cohesion emerged as a protective factor for those with high school or more education but a risk factor for those with less than a high school education.

The current findings add to the growing body of literature finding mixed support for neighborhood social cohesion on CVH in both cross-sectional and longitudinal studies. In our study, neighborhood social cohesion was not associated with ideal/intermediate LS7 over time. Unger and colleagues did not find associations between higher neighborhood social cohesion and ideal CVH in their cross-sectional study with MESA participants [33]. However, in cross-sectional analyses of the Morehouse-Emory Cardiovascular study of Black adults in a southern US metropolitan area identified neighborhood social cohesion as being associated positively with ideal LS7 and some LS7 components [18]. Results from the Health and Retirement Study (HRS) also provide some evidence that neighborhood social cohesion is associated positively with a summary index of cardiometabolic health at four-year follow-up [41]. However, one potential limitation of this HRS analysis is that it included physical activity as a mediator instead of in a summary measure of CVH. In a separate longitudinal analysis of HRS data, the findings did not support any associations of neighborhood social cohesion with smoking, physical activity or cardiovascular-related diseases at four-year follow-up [19]. Perhaps these differences in findings between HRS analyses are due in part, to different operationalizations of the CVH outcomes [19]. Despite the equivocal findings, our results align with the longitudinal HRS findings with racially and ethnically diverse older adults suggesting that neighborhood social cohesion may not be associated with CVH outcomes over time [19].

Although we did not find support for the role of neighborhood social cohesion as a resilience resource on LS7 over time, cross-sectional study findings suggest that there are relationships with LS7 components. Research with Asian American subgroups suggests that neighborhood social cohesion is associated with engaging in some CVH screening behaviors across genders, smoking among men, and with lower odds of hypertension and high BMI among women.[42–45] Results from systematic review studies also indicate that neighborhood social cohesion is associated with physical activity and there is some evidence for associations with weight status [17]. These cross-sectional studies suggest that the presence and/or strength of relationships between neighborhood social cohesion and CVH components may differ. However, findings in support of a relationship between neighborhood social cohesion and LS7 or its components are equivocal.

One reason for the equivocal findings may be that researchers conceptualize and measure neighborhood social cohesion in different ways [17]. Neighborhood social cohesion is multicomponent and the field could be strengthened by conceptualizing it more comprehensively, examining multiple components in-depth and harmonizing measures across studies [17]. Additionally, some researchers caution that neighborhood social cohesion could have unintended negative consequences (e.g., taxing of neighbors’ resources and transmission of unhealthy norms) on health [46]. This concern is highlighted in our work suggesting that higher neighborhood social cohesion is associated with worse CVH among those with lower education. However, it is protective among those with higher education. This finding suggests that neighborhood social cohesion could be a protective resource for some subgroups, but may not be a resilience resource for people experiencing adversities. Additionally, CVH is measured inconsistently or only single components of CVH are assessed in studies. These approaches limit understanding of the relationship between neighborhood social cohesion and overall CVH profiles. Additional longitudinal studies examining more comprehensive measures of neighborhood social cohesion in relation to LS7 outcomes and that assess for effect modification of adversities are warranted.

Although informative, this study is not without some limitations. The neighborhood social cohesion measure, while used extensively in prior studies, is limited in scope because it does not fully capture the social inclusion and social capital components of neighborhood social cohesion. While we assessed neighborhood social cohesion at the individual level and as tertiles, both of which are common, tertiles are sample dependent and may not truly reflect levels of high, medium or low in the general population [19]. Future studies could use aggregate measures of perceived and/or objective measures of neighborhood social cohesion as tertiles or continuous measures. To our knowledge, there are no established cut-points for the measure used. Also, we used census tracts as proxies for neighborhood environments; these may not map onto study participants’ perceptions of their neighborhoods. We did not account for participants moving to different census tracts after Exam 1. However, comparing the census tract information available at Exams 1 and 2 in the primary analysis, 89.3% of the included MASALA and MESA participants resided in the same census tract at Exam 2 as they did at Exam (1) In the secondary analysis, 87.7% of the included JHS, MASALA, and MESA participants resided in the same census tract at Exams 1 and (2) Also, the exclusion of participants and visits with unavailable information may have resulted in selection bias. However, this potential selection bias was minimized via regression adjustment. Our study findings may not be generalizable to other populations with a different distribution of EMMs (e.g., education). Last, 95.7% (5,822/6,086) of the outcomes assessed at the first visit were measured concurrently with the exposure. However, findings did not differ meaningfully between visits 1 and 2, which suggests that reverse causation may have had minimal impact. Despite these limitations, this study is strengthened by the diverse populations included, available robust data and longitudinal analysis with appropriate temporal ordering of variables.

## Conclusion

In conclusion, this study did not find much evidence of an overall relationship between neighborhood social cohesion and CVH. We also found mixed support for some psychosocial stressors as effect modifiers. Future studies should examine neighborhood social cohesion more comprehensively, assess it over longer periods of time and in socioeconomically diverse and nationally representative cohort studies. As well, future studies should assess for effect modification and include and examine composite LS7 over time, and only include prospective LS7 measures.

## Electronic supplementary material

Below is the link to the electronic supplementary material.

Supplementary Material 1

## Data Availability

Data may be obtained from a third party and are not publicly available. The datasets supporting the conclusions of this article originate from three separate third party sources. Restrictions apply to the availability of the data, which were used under fully executed Data Materials Distribution Agreement and Data Use Agreements. Data from the Jackson Heart Study is available with a request to access at https://www.jacksonheartstudy.org/Research/Study-Data/Data-Access. Data from the Multi-Ethnic Study of Atherosclerosis is available with a request to access at https://www.mesa-nhlbi.org/Publications.aspx. Data from the Mediators of Atherosclerosis among South Asians Living in America Study is available with permission at https://www.masalastudy.org/for-researchers.

## List of abbreviations

aPRs: adjusted prevalence ratios.
BMI: body mass index.
CVH: cardiovascular health.
CVD: cardiovascular disease.
HRS: Health and Retirement Study.
IRB: Institutional Review Board.
JHS: Jackson Heart Study.
LS7: Life’s Simple 7.
MASALA: Mediators of Atherosclerosis in South Asians Living in America.
MESA: Multi-Ethnic study of Atherosclerosis.
US: United States.
