# Supplementary material for: Examining relationships between perceived neighborhood social cohesion and ideal cardiovascular health and whether psychosocial stressors modify observed relationships among JHS, MESA, and MASALA participants
Source: BMC Public Health. 2022 Oct 11;22:1890. doi: 10.1186/s12889-022-14270-x (PMC9552445; doi:10.1186/s12889-022-14270-x)
Supplement: Supplementary file 1 — Supplementary Material 1 [file 12889_2022_14270_MOESM1_ESM.docx]

**Additional File**

Filename: Additional file 1

File format: .doc

Title of data: Supplemental Files

Description of data: Supplemental figures and data tables presenting results of primary and secondary analysis results, including by visit and cohort

**Supplemental Figure 1.** Causal directed acyclic graph showing relationships between the study exposure (neighborhood social cohesion), the study outcome (ideal cardiovascular health), the study covariates (confounders, sources of selection bias, and potential effect modifiers), and study restrictions (“Selected”). A box around “Selected” represents conditioning due to the exclusion of persons or visits from the analysis.

**Supplemental Figure 2.** Exclusion criteria to identify 6,086 MASALA and MESA participants included in the primary analysis

Abbreviations: CVH= cardiovascular health, JHS= Jackson Heart Study, LS7= Life’s Simple 7, MASALA= Mediators of Atherosclerosis Among South Asians Living in America, MESA=Multi-Ethnic Study of Atherosclerosis.

* This exclusion step resulted in the exclusion of all remaining JHS participants because neighborhood social cohesion was assessed during the third annual follow-up interview, which was after when LS7 metrics for ideal CVH was assessed.

**Supplemental Figure 3.** Exclusion criteria to identify 7,291 JHS, MASALA, and MESA participants included in the secondary analysis

Abbreviations: CVH= cardiovascular health, JHS= Jackson Heart Study, LS7= Life’s Simple 7, MASALA= Mediators of Atherosclerosis Among South Asians Living in America, MESA=Multi-Ethnic Study of Atherosclerosis.

**Supplemental Table 1.** Characteristics of JHS, MASALA, and MESA participants*

| **Characteristics** | **Included (n =7,291)** | | | **Excluded (n =456)** | | **P-value^**^** |
| --- | --- | --- | --- | --- | --- | --- |
|  | **N** | **%** | | **N** | **%** |  |
| **Neighborhood social cohesion**^†^ **at**  **MASALA/MESA Exam 1 or JHS AF3** | | | |  |  |  |
| Low | 2,334 | 32.0 | | 126 | 27.6 | <0.01 |
| Medium | 2,810 | 38.5 | | 147 | 32.2 |  |
| High | 2,147 | 29.5 | | 183 | 40.1 |  |
| **Age**^‡^ **(years) at Exam 1** | 59 (51-68) | | | 58 (47.5-67) | | <0.01 |
| **Sex/gender at Exam 1** |  |  | |  |  |  |
| Female | 3,935 | 54.0 | | 276 | 60.5 | <0.01 |
| Male | 3,356 | 46.0 | | 180 | 39.5 |  |
| **Self-reported race/ethnicity at Exam 1** | |  | |  |  |  |
| White non-Hispanic | 2,252 | 30.9 | | 36 | 7.9 | <0.01 |
| Asian | 1,071 | 14.7 | | 25 | 5.5 |  |
| African American | 2,727 | 37.4 | | 379 | 83.1 |  |
| Hispanic | 1,241 | 17.0 | | 16 | 3.5 |  |
| **Nativity at Exam 1** |  |  | |  |  |  |
| Other | 2,114 | 29.0 | | 46 | 10.1 | <0.01 |
| U.S.-born | 5,177 | 71.0 | | 410 | 89.9 |  |
| **Region at Exam 1** |  |  | |  |  |  |
| West | 1,107 | 15.2 | | 19 | 4.2 | <0.01 |
| South | 2,154 | 29.5 | | 362 | 79.4 |  |
| Midwest | 2,318 | 31.8 | | 22 | 4.8 |  |
| Northeast | 1,712 | 23.5 | | 53 | 11.6 |  |
| **Marital status at Exam 1** |  |  | |  |  |  |
| Never married, separated/divorced, widowed | 2,651 | 36.4 | | 198 | 43.4 | <0.01 |
| Married | 4,640 | 63.6 | | 258 | 56.6 |  |
| **Self-rated health^§^ at Exam 1** |  |  | |  |  |  |
| Not good | 822 | 11.3 | | 121 | 26.5 | <0.01 |
| Good | 6,469 | 88.7 | | 335 | 73.5 |  |
| **Health insurance at Exam 1** |  |  | |  |  |  |
| None | 658 | 9.0 | | 57 | 12.5 | 0.01 |
| Public or Private | 6,633 | 91.0 | | 399 | 87.5 |  |
| **Self-history of CVD and stroke at Exam 1** | | | |  |  |  |
| No | 7,220 | 99.0 | | 415 | 91.0 | <0.01 |
| Yes | 71 | 1.0 | | 41 | 9.0 |  |
| **Family history of CVD and stroke at Exam 1** | | | |  |  |  |
| No | 3,154 | 43.3 | | 214 | 46.9 | 0.13 |
| Yes | 4,137 | 56.7 | | 242 | 53.1 |  |
| **Education at Exam 1** |  |  | |  |  |  |
| Less than high school | 1,029 | 14.1 | | 76 | 16.7 | 0.20 |
| High school or some college | 3,235 | 44.4 | | 206 | 45.2 |  |
| College degree or more | 3,027 | 41.5 | | 174 | 38.2 |  |
| **Employment at Exam 1** |  |  | |  |  |  |
| Unemployed | 3,399 | 46.6 | | 242 | 53.1 | <0.01 |
| Employed | 3,892 | 53.4 | | 214 | 46.9 |  |
| **Income at Exam 1** |  |  | |  |  |  |
| $0-$19,999 | 1,519 | 20.8 | | 144 | 31.6 | <0.01 |
| $20,000-$49,999 | 2,618 | 35.9 | | 156 | 34.2 |  |
| $50,000+ | 3,154 | 43.3 | | 156 | 34.2 |  |
| **Anger**^†^ **at Exam 1** |  |  | |  |  |  |
| Low | 2,763 | 37.9 | | 146 | 32.0 | <0.01 |
| Medium | 2,369 | 32.5 | | 117 | 25.7 |  |
| High | 2,159 | 29.6 | | 193 | 42.3 |  |
| **Depressive symptoms at Exam 1** |  |  | |  |  |  |
| No | 6,304 | 86.5 | | 355 | 77.9 | <0.01 |
| Yes | 987 | 13.5 | | 101 | 22.2 |  |
| **Chronic stress**^†^ **at Exam 1** |  |  | |  |  |  |
| Low | 3,232 | 44.3 | | 112 | 24.6 | <0.01 |
| Medium | 2,428 | 33.3 | | 182 | 39.9 |  |
| High | 1,631 | 22.4 | | 162 | 35.5 |  |
| **Discrimination**^†^ **at Exam 1** |  |  | |  |  |  |
| Low | 2,648 | 36.3 | | 148 | 32.5 | <0.01 |
| Medium | 2,439 | 33.5 | | 137 | 30.0 |  |
| High | 2,204 | 30.2 | | 171 | 37.5 |  |
| **Neighborhood deprivation**^†^ **at Exam 1** | | |  |  |  |  |
| Low | 2,059 | 28.2 | | 234 | 51.3 | <0.01 |
| Medium | 2,557 | 35.1 | | 124 | 27.2 |  |
| High | 2,675 | 36.7 | | 98 | 21.5 |  |
| **Neighborhood safety at Exam 1** | |  | |  |  |  |
| Safe | 5,894 | 80.8 | | 294 | 64.5 | <0.01 |
| Not safe | 1,397 | 19.2 | | 162 | 35.5 |  |
| **Social support at Exam 1** |  |  | |  |  |  |
| Not high | 2,596 | 35.6 | | 151 | 33.1 | 0.28 |
| High | 4,695 | 64.4 | | 305 | 66.9 |  |

^*^ Assessed at the exam or interview that assessed the exposure (i.e., neighborhood social cohesion) or exams concurrent with or before exposure assessment comparing the included and a subset of the excluded participants (i.e., those without all four biological LS7 metrics assessed at least once at or after exposure assessment) in the secondary analyses.

^**^ Pearson’s χ^2^-test or Wilcoxon-Mann-Whitney test.

^†^ Tertiles are not 33% due to ties at boundaries and participants with the same value were included in the same category.

^‡^ Median **(**25^th^ percentile-75^th^ percentile).

**^§^** Binary variable for self-rated health was used to indicate ‘Good’ and ‘Not good’ categories due to the harmonization of different self-rated health measures across JHS, MESA, and MASALA cohorts.

Abbreviations: CVD, cardiovascular disease; AF3, Third Annual Follow-up Interview; JHS, Jackson Heart Study; MESA, Multi-Ethnic Study of Atherosclerosis.

**Supplemental Table 2**. Assessment of effect measure modification of adjusted prevalence ratios^*^ (aPR) using four biological Life’s Simple 7 (secondary analysis sample)

| **Psychosocial risk measure**  **(Potential effect measure modifier)** | **High versus low neighborhood social cohesion and ideal or intermediate (but no poor) metrics by level of psychosocial risk measure** | | **Medium versus low neighborhood social cohesion and ideal or intermediate (but no poor) metrics by level of psychosocial risk measure** | | **p**^†^ |
| --- | --- | --- | --- | --- | --- |
|  | **aPR** | **95% CI** | **aPR** | **95% CI** |  |
| **Education at Exam 1** |  |  |  |  |  |
| College degree or more | 1.05 | (0.96-1.16) | 1.00 | (0.91-1.09) | 0.09 |
| High school or some college | 1.04 | (0.93-1.15) | 1.05 | (0.96-1.14) |  |
| Less than high school | 0.79 | (0.65-0.97) | 0.94 | (0.79-1.11) |  |
| **Employment at Exam 1** |  |  |  |  |  |
| Employed | 1.05 | (0.96-1.14) | 1.00 | (0.92-1.08) | 0.28 |
| Unemployed | 0.97 | (0.88-1.07) | 1.02 | (0.93-1.11) |  |
| **Income at Exam 1** |  |  |  |  |  |
| $50,000+ | 1.13 | (1.03-1.23) | 1.04 | (0.95-1.14) | 0.02 |
| $20,000-$49,999 | 0.91 | (0.81-1.02) | 0.98 | (0.89-1.08) |  |
| $0-$19,999 | 0.90 | (0.76-1.06) | 1.00 | (0.88-1.14) |  |
| **Anger at Exam 1** |  |  |  |  |  |
| Low | 1.08 | (0.98-1.18) | 1.07 | (0.98-1.18) | 0.39 |
| Medium | 1.01 | (0.90-1.13) | 0.98 | (0.89-1.09) |  |
| High | 0.95 | (0.84-1.06) | 0.96 | (0.86-1.07) |  |
| **Depressive symptoms at Exam 1** |  |  |  |  |  |
| No | 1.03 | (0.96-1.10) | 1.00 | (0.94-1.07) | 0.16 |
| Yes | 0.88 | (0.72-1.07) | 1.04 | (0.89-1.22) |  |
| **Chronic stress at Exam 1** |  |  |  |  |  |
| Low | 1.05 | (0.96-1.16) | 1.07 | (0.99-1.15) | 0.34 |
| Medium | 0.98 | (0.87-1.11) | 0.97 | (0.87-1.08) |  |
| High | 0.98 | (0.82-1.16) | 0.92 | (0.79-1.07) |  |
| **Discrimination at Exam 1** |  |  |  |  |  |
| Low | 0.95 | (0.86-1.05) | 1.01 | (0.93-1.10) | 0.06 |
| Medium | 0.98 | (0.89-1.09) | 0.98 | (0.89-1.07) |  |
| High | 1.17 | (1.03-1.32) | 1.03 | (0.92-1.16) |  |
| **Neighborhood deprivation at Exam 1** | |  |  |  |  |
| Low | 0.90 | (0.77-1.07) | 0.98 | (0.87-1.11) | 0.42 |
| Medium | 1.01 | (0.90-1.13) | 0.98 | (0.90-1.08) |  |
| High | 1.08 | (0.98-1.18) | 1.05 | (0.96-1.15) |  |
| **Neighborhood safety at Exam 1** | |  |  |  |  |
| Safe | 1.01 | (0.94-1.09) | 1.01 | (0.95-1.08) | 0.80 |
| Not safe | 1.05 | (0.89-1.24) | 0.99 | (0.84-1.15) |  |

Note: Each modified Poisson regression model accounted for clustering within neighborhood (i.e., census tract at Exam 1).

Note: Assessments for the relationship between neighborhood social cohesion at Exam 1 in MASALA/MESA and the Third Annual Follow-up Interview in JHS and ideal or intermediate (but no poor) metrics versus at least 1 poor metric using four biological Life’s Simple 7 metrics (BMI, blood pressure, cholesterol, and glucose) by levels of psychosocial risk measures among participants included in the secondary analysis sample (N=7,291).

^*^ Adjusted for visit, age, sex/gender, race, nativity, geographic region, marital status, self-rated health, insurance, family CVD history, social support, education, income, employment, anger, depressive symptoms, chronic stress, discrimination, neighborhood deprivation, and neighborhood safety.

^†^ Global chi-squared test provided p-values to indicate whether at least one of the coefficients of the product terms between neighborhood social cohesion and psychosocial risk were different from zero.

**Supplemental Table 3.** Assessment of effect measure modification of adjusted prevalence ratios^*^ (aPR) for cardiovascular risk (secondary analysis sample)

| **Psychosocial risk measure**  **(Potential effect measure modifier)** | **High versus low neighborhood social cohesion and lower cardiovascular risk (0-1 poor metrics) by level of psychosocial risk measure** | | **Medium versus low neighborhood social cohesion lower cardiovascular risk (0-1 poor metrics) by level of psychosocial risk measure** | | **p**^†^ |
| --- | --- | --- | --- | --- | --- |
|  | **aPR** | **95% CI** | **aPR** | **95% CI** |  |
| **Education at Exam 1** |  |  |  |  |  |
| College degree or more | 1.01 | (0.98-1.04) | 1.00 | (0.97-1.04) | 0.32 |
| High school or some college | 0.99 | (0.95-1.04) | 1.01 | (0.97-1.05) |  |
| Less than high school | 0.93 | (0.85-1.02) | 1.01 | (0.96-1.07) |  |
| **Employment at Exam 1** |  |  |  |  |  |
| Employed | 1.01 | (0.98-1.04) | 1.01 | (0.98-1.05) | 0.34 |
| Unemployed | 0.97 | (0.94-1.01) | 1.00 | (0.97-1.03) |  |
| **Income at Exam 1** |  |  |  |  |  |
| $50,000+ | 1.02 | (0.98-1.05) | 1.01 | (0.98-1.04) | 0.09 |
| $20,000-$49,999 | 1.00 | (0.95-1.05) | 1.01 | (0.97-1.06) |  |
| $0-$19,999 | 0.92 | (0.85-0.99) | 0.99 | (0.95-1.04) |  |
| **Anger at Exam 1** |  |  |  |  |  |
| Low | 1.01 | (0.97-1.05) | 1.01 | (0.97-1.05) | 0.40 |
| Medium | 0.99 | (0.95-1.03) | 0.99 | (0.95-1.02) |  |
| High | 0.97 | (0.92-1.02) | 1.02 | (0.98-1.06) |  |
| **Depressive symptoms at Exam 1** |  |  |  |  |  |
| No | 1.00 | (0.97-1.02) | 1.00 | (0.98-1.03) | 0.44 |
| Yes | 0.97 | (0.90-1.06) | 1.03 | (0.97-1.10) |  |
| **Chronic stress at Exam 1** |  |  |  |  |  |
| Low | 1.01 | (0.98-1.05) | 1.01 | (0.98-1.04) | 0.55 |
| Medium | 0.98 | (0.94-1.03) | 1.01 | (0.97-1.05) |  |
| High | 0.96 | (0.91-1.02) | 0.99 | (0.94-1.04) |  |
| **Discrimination at Exam 1** |  |  |  |  |  |
| Low | 0.98 | (0.94-1.02) | 0.99 | (0.96-1.02) | 0.64 |
| Medium | 1.00 | (0.96-1.04) | 1.00 | (0.97-1.04) |  |
| High | 1.01 | (0.96-1.06) | 1.03 | (0.99-1.08) |  |
| **Neighborhood deprivation at Exam 1** | |  |  |  |  |
| Low | 0.98 | (0.92-1.03) | 0.97 | (0.92-1.02) | 0.44 |
| Medium | 0.99 | (0.94-1.04) | 1.01 | (0.97-1.06) |  |
| High | 1.01 | (0.98-1.05) | 1.02 | (1.00-1.05) |  |
| **Neighborhood safety at Exam 1** | |  |  |  |  |
| Safe | 1.00 | (0.97-1.03) | 1.01 | (0.98-1.04) | 0.25 |
| Not safe | 0.94 | (0.87-1.01) | 0.99 | (0.94-1.05) |  |

Note: Each modified Poisson regression model accounted for clustering within neighborhood (i.e., census tract at Exam 1).

Assessment of effect measure modification of (aPR) for the relationship between neighborhood social cohesion at Exam 1 in MASALA/MESA and the Third Annual Follow-up Interview in JHS and lower cardiovascular risk (0-1 poor metrics) versus non-lower cardiovascular risk (2-4 poor metrics) using four biological Life’s Simple 7 metrics (BMI, blood pressure, cholesterol, and glucose) by levels of psychosocial risk measures among participants included in the secondary analysis sample (N=7,291).

^*^ Adjusted for visit, age, sex/gender, race, nativity, geographic region, marital status, self-rated health, insurance, family CVD history, social support, education, income, employment, anger, depressive symptoms, chronic stress, discrimination, neighborhood deprivation, and neighborhood safety.

^†^ Global chi-squared test provided p-values to indicate whether at least one of the coefficients of the product terms between neighborhood social cohesion and psychosocial risk were different from zero.

**Supplemental Table 4**. Cohort-stratified^*^ adjusted^†^ prevalence ratios (aPR) for ideal or intermediate (versus poor) CVH using Life’s Simple 7

| **Outcome** | **Neighborhood social cohesion and visit product term in outcome model** | | **High versus low**  **neighborhood social cohesion**  **aPR (95% CI)** | | **Medium versus low**  **neighborhood social cohesion**  **aPR (95% CI)** | |
| --- | --- | --- | --- | --- | --- | --- |
|  |  |  | **MASALA**  **(N =371)** | **MESA**  **(N =5,715)** | **MASALA**  **(N =371)** | **MESA**  **(N =5,715)** |
| Ideal or intermediate (versus poor) CVH | No product term in model | | 1.15 (0.98-1.35) | 1.01 (0.97-1.06) | 1.00 (0.84-1.20) | 1.02 (0.98-1.06) |
|  | Product term is present^‡^ | Visit 1 | 1.14 (0.98-1.34) | 1.01 (0.97-1.05) | 1.00 (0.84-1.20) | 1.03 (0.99-1.07) |
|  |  | Visit 2 | 1.46 (0.62-3.44) | 1.02 (0.97-1.08) | 1.13 (0.36-3.49) | 1.02 (0.96-1.07) |

Note: Each modified Poisson regression model accounted for clustering within neighborhood (i.e., census tract at Exam 1).

^*^ JHS was not included in the primary analysis.

^†^ Adjusted for visit, age, sex/gender, race, nativity, geographic region, marital status, self-rated health, insurance, family history of CVD and stroke, social support education, income, employment, anger, depressive symptoms, chronic stress, discrimination, neighborhood deprivation, and neighborhood safety. In MASALA, race and region were excluded from the model because data were from South Asians residing in one geographic location.

^‡^ Neighborhood social cohesion and visit product term coefficients in MASALA: 0.12, 0.25, p=0.80 and in MESA: -0.01, 0.02, p=0.62.

**Supplemental Table 5**. Cohort-stratified adjusted^*^ prevalence ratios (aPR) using four biological Life’s Simple 7 measures

| **Outcome** | **Neighborhood social cohesion and visit product term in outcome model** | | **High versus low**  **neighborhood social cohesion**  **aPR (95% CI)** | | | **Medium versus low**  **neighborhood social cohesion**  **aPR (95% CI)** | | |
| --- | --- | --- | --- | --- | --- | --- | --- | --- |
|  |  |  | **JHS**  **(N=1,309)** | **MASALA**  **(N =380)** | **MESA**  **(N =5,602)** | **JHS**  **(N=1,309)** | **MASALA**  **(N =380)** | **MESA**  **(N =5,602)** |
| Ideal or intermediate (but no poor) metrics | No product term in model | | 1.11  (0.84-1.47) | 1.10  (0.85-1.41) | 1.01  (0.94-1.09) | 0.82  (0.61-1.10) | 0.90  (0.69-1.17) | 1.03  (0.96-1.09) |
|  | Product term is present^†^ | Visit 1 | 1.08  (0.82-1.42) | 1.12  (0.87-1.45) | 1.02  (0.94-1.10) | 0.82  (0.61-1.09) | 0.90  (0.70-1.17) | 1.01  (0.95-1.08) |
|  |  | Visit 2 | 2.16  (0.53-8.90) | 0.53  (0.13-2.17) | 1.01  (0.92-1.10) | 0.76  (0.10-5.98) | 0.73  (0.10-5.53) | 1.04  (0.96-1.13) |
| Lower cardiovascular risk (0-1 poor metrics) | No product term in model | | 0.94  (0.85-1.04) | 1.02  (0.94-1.11) | 1.00  (0.97-1.03) | 0.93  (0.84-1.03) | 1.00  (0.91-1.10) | 1.01  (0.99-1.04) |
|  | Product term is present^‡^ | Visit 1 | 0.95  (0.86-1.06) | 1.01  (0.93-1.09) | 1.00  (0.97-1.03) | 0.94  (0.85-1.04) | 0.99  (0.90-1.08) | 1.03  (1.00-1.06) |
|  |  | Visit 2 | 0.81  (0.57-1.16) | 1.40  (0.71-2.77) | 1.00  (0.97-1.03) | 0.87  (0.58-1.31) | 1.47  (0.77-2.81) | 0.99  (0.96-1.02) |

Note: Each modified Poisson regression model accounted for clustering within neighborhood (i.e., census tract at Exam 1).

Note: Cohort-stratified adjusted^*^ prevalence ratios (aPR) for ideal or intermediate (but no poor) metrics versus at least 1 poor metric and lower cardiovascular risk (0-1 poor metrics) versus non-lower CV risk (2-4 poor metrics) using four biological Life’s Simple 7 measures (BMI, blood pressure, cholesterol, and glucose) assessed over two visits.

^*^ Adjusted for visit, age, sex/gender, race, nativity, geographic region, marital status, self-rated health, insurance, self-history of CVD and stroke, family history of CVD and stroke, social support, education, income, employment, anger, depressive symptoms, chronic stress, discrimination, neighborhood deprivation, and neighborhood safety. In JHS, race, nativity, and geographic region were excluded from the model because data were from African American residing in one geographic region at Exam 1. In MASALA, race, region, and self-history of CVD and stroke were excluded from the model because data were from South Asians residing in one geographic location and participants were free of CVD at study enrollment. In MESA, self-history of CVD was excluded from the model because all participants were free of CVD at study enrollment.

^†^ Neighborhood social cohesion and visit product term coefficients in JHS: -0.07, 0.69, p=0.32; in MASALA: -0.21, -0.75, p=0.62; and in MESA: 0.02, -0.006, p=0.75.

^‡^ Neighborhood social cohesion and visit product term coefficients in JHS: -0.08, -0.16, p=0.68; in MASALA: 0.39, 0.33, p=0.39 and in MESA: -0.03, 0.0007, p=0.06.

**Supplemental Table 6.** Assessment of effect measure modification of adjusted prevalence ratios^*^ (aPR) in the primary analysis sample

| **Psychosocial risk measures**  **(Potential effect measure modifier)** | **High versus low neighborhood social cohesion and ideal or intermediate versus poor CVH by visit and level of psychosocial risk measure** | | | | | | | | **Medium versus low neighborhood social cohesion and ideal or intermediate versus poor CVH by visit and level of psychosocial risk measure** | | | | | | | | **p**^†^ | |
| --- | --- | --- | --- | --- | --- | --- | --- | --- | --- | --- | --- | --- | --- | --- | --- | --- | --- | --- |
|  | **Visit 1** | | | **Visit 2** | | | | | **Visit 1** | | | | **Visit 2** | | | |  | |
|  | **aPR** | **95% CI** | | | **aPR** | | **95% CI** | | **aPR** | | **95% CI** | | **aPR** | | **95% CI** | |  | |
| **Education at Exam 1** |  |  | | |  | |  | |  | |  | |  | |  | |  | |
| College degree or more | 1.02 | (0.97-1.07) | | | 1.09 | | (1.02-1.17) | | 1.02 | | (0.97-1.07) | | 1.08 | | (1.01-1.15) | | 0.02 | |
| High school or some college | 1.08 | (1.01-1.16) | | | 1.00 | | (0.90-1.11) | | 1.04 | | (0.98-1.12) | | 1.00 | | (0.92-1.09) | |  |  |
| Less than high school | 0.74 | (0.63-0.87) | | | 0.83 | | (0.67-1.02) | | 1.02 | | (0.92-1.14) | | 0.90 | | (0.77-1.05) | |  |  |
| **Employment at Exam 1** |  |  | | |  | |  | |  | |  | |  | |  | |  | |
| Employed | 1.02 | (0.96-1.08) | | | 1.01 | | (0.94-1.10) | | 1.03 | | (0.97-1.08) | | 1.01 | | (0.93-1.09) | | 0.74 | |
| Unemployed | 0.99 | (0.93-1.05) | | | 1.03 | | (0.94-1.12) | | 1.02 | | (0.97-1.08) | | 1.03 | | (0.95-1.12) | |  |  |
| **Income at Exam 1** |  |  | | |  | |  | |  | |  | |  | |  | |  | |
| $50,000+ | 1.02 | (0.97-1.08) | | | 1.04 | | (0.97-1.12) | | 1.02 | | (0.97-1.08) | | 1.00 | | (0.93-1.07) | | 0.76 | |
| $20,000-$49,999 | 1.00 | (0.93-1.08) | | | 0.97 | | (0.87-1.08) | | 1.02 | | (0.95-1.08) | | 1.00 | | (0.92-1.09) | |  |  |
| $0-$19,999 | 0.96 | (0.85-1.08) | | | 1.01 | | (0.86-1.19) | | 1.05 | | (0.96-1.14) | | 1.11 | | (0.99-1.24) | |  |  |
| **Anger at Exam 1** |  |  | | |  | |  | |  | |  | |  | |  | |  | |
| Low | 1.01 | (0.94-1.08) | | | 1.06 | | (0.97-1.16) | | 1.02 | | (0.96-1.08) | | 1.07 | | (0.98-1.16) | | 0.66 | |
| Medium | 1.01 | (0.94-1.08) | | | 1.02 | | (0.94-1.11) | | 1.00 | | (0.95-1.06) | | 0.98 | | (0.91-1.07) | |  |  |
| High | 0.99 | (0.91-1.08) | | | 0.97 | | (0.87-1.08) | | 1.06 | | (0.98-1.14) | | 1.00 | | (0.91-1.10) | |  |  |
| **Depressive symptoms at Exam 1** |  |  | | |  | |  | |  | |  | |  | |  | |  | |
| No | 1.01 | (0.96-1.05) | | | 1.03 | | (0.97-1.09) | | 1.02 | | (0.98-1.06) | | 1.02 | | (0.97-1.08) | | 0.60 | |
| Yes | 0.97 | (0.83-1.14) | | | 0.94 | | (0.77-1.15) | | 1.06 | | (0.94-1.19) | | 0.97 | | (0.84-1.13) | |  |  |
| **Chronic stress at Exam 1** | | |  | | |  | |  | |  | |  | |  | |  | |  |
| Low | 1.03 | (0.98-1.10) | | | 0.99 | | (0.91-1.07) | | 1.05 | | (1.00-1.11) | | 1.01 | | (0.95-1.08) | | 0.32 | |
| Medium | 0.96 | (0.90-1.03) | | | 1.05 | | (0.95-1.17) | | 0.98 | | (0.92-1.05) | | 1.02 | | (0.93-1.13) | |  |  |
| High | 1.01 | (0.91-1.12) | | | 1.06 | | (0.93-1.22) | | 1.01 | | (0.92-1.12) | | 1.01 | | (0.89-1.15) | |  |  |
| **Discrimination at Exam 1** | | |  | | |  | |  | |  | |  | |  | |  | |  |
| Low | 0.96 | (0.90-1.02) | | | 0.97 | | (0.89-1.06) | | 1.00 | | (0.94-1.06) | | 0.97 | | (0.89-1.04) | | 0.65 | |
| Medium | 1.01 | (0.95-1.08) | | | 1.04 | | (0.94-1.15) | | 0.99 | | (0.94-1.06) | | 1.05 | | (0.96-1.14) | |  |  |
| High | 1.08 | (0.99-1.17) | | | 1.08 | | (0.98-1.18) | | 1.09 | | (1.02-1.18) | | 1.06 | | (0.96-1.16) | |  |  |
| **Neighborhood deprivation at Exam 1** | | | | | |  | |  | |  | |  | |  | |  | |  |
| Low | 0.95 | (0.85-1.07) | | | 1.04 | | (0.90-1.21) | | 1.01 | | (0.93-1.10) | | 1.00 | | (0.89-1.13) | | 0.27 | |
| Medium | 1.01 | (0.94-1.08) | | | 1.00 | | (0.89-1.12) | | 1.02 | | (0.96-1.10) | | 1.06 | | (0.96-1.17) | |  |  |
| High | 1.04 | (0.98-1.10) | | | 1.01 | | (0.94-1.08) | | 1.04 | | (0.99-1.10) | | 0.97 | | (0.91-1.04) | |  |  |
| **Neighborhood safety at Exam 1** | | | | | |  | |  | |  | |  | |  | |  | |  |
| Safe | 1.01 | (0.96-1.05) | | | 0.99 | | (0.93-1.05) | | 1.03 | | (0.99-1.08) | | 0.99 | | (0.93-1.05) | | 0.02 | |
| Not safe | 1.07 | (0.95-1.21) | | | 1.11 | | (0.94-1.32) | | 0.99 | | (0.89-1.09) | | 1.16 | | (1.03-1.30) | |  |  |

Note: Each modified Poisson regression model accounted for clustering within neighborhood (i.e., census tract at Exam 1).

Assessment of effect measure modification of adjusted prevalence ratios^*^ (aPR) for each visit and for ideal or intermediate (versus poor) CVH using Life’s Simple 7 metrics levels of psychosocial risk measures and social support among participants included in the primary analysis sample (N=6,086).

^*^ Adjusted for visit, age, sex/gender, race, nativity, geographic region, marital status, self-rated health, insurance, self-history of CVD and stroke, family history of CVD and stroke, social support, education, income, employment, anger, depressive symptoms, chronic stress, discrimination, neighborhood deprivation, neighborhood safety, and all possible product terms between visit, neighborhood social cohesion, and psychosocial risk.

^†^ P-values were obtained from a global chi-squared test.

**Supplemental Table 7.** Assessment of effect measure modification of adjusted prevalence ratios* (aPR) for each visit (secondary analysis sample)

| **Psychosocial risk measures**  **(Potential effect measure modifier)** | **High versus low neighborhood social cohesion and ideal or intermediate (but no poor) metrics by visit and level of psychosocial risk measure** | | | | | | | | **Medium versus low neighborhood social cohesion and ideal or intermediate (but no poor) metrics by visit and level of psychosocial risk measure** | | | | | | | | **p**^†^ | |
| --- | --- | --- | --- | --- | --- | --- | --- | --- | --- | --- | --- | --- | --- | --- | --- | --- | --- | --- |
|  | **Visit 1** | | | **Visit 2** | | | | | **Visit 1** | | | | **Visit 2** | | | |  | |
|  | **aPR** | **95% CI** | | | **aPR** | | **95% CI** | | **aPR** | | **95% CI** | | **aPR** | | **95% CI** | |  | |
| **Education at Exam 1** |  |  | | |  | |  | |  | |  | |  | |  | |  | |
| College degree or more | 1.08 | (0.98-1.19) | | | 1.01 | | (0.89-1.14) | | 0.98 | | (0.89-1.07) | | 1.04 | | (0.92-1.17) | | 0.30 | |
| High school or some college | 1.01 | (0.90-1.13) | | | 1.08 | | (0.94-1.25) | | 1.04 | | (0.94-1.14) | | 1.06 | | (0.95-1.19) | |  |  |
| Less than high school | 0.82 | (0.66-1.02) | | | 0.74 | | (0.54-1.00) | | 0.94 | | (0.78-1.13) | | 0.94 | | (0.75-1.17) | |  |  |
| **Employment at Exam 1** |  |  | | |  | |  | |  | |  | |  | |  | |  | |
| Employed | 1.05 | (0.96-1.15) | | | 1.03 | | (0.92-1.15) | | 0.98 | | (0.91-1.06) | | 1.03 | | (0.92-1.14) | | 0.99 | |
| Unemployed | 0.98 | (0.88-1.09) | | | 0.97 | | (0.85-1.10) | | 1.00 | | (0.91-1.10) | | 1.05 | | (0.93-1.18) | |  |  |
| **Income at Exam 1** |  |  | | |  | |  | |  | |  | |  | |  | |  | |
| $50,000+ | 1.13 | (1.03-1.24) | | | 1.12 | | (1.00-1.26) | | 1.03 | | (0.94-1.13) | | 1.06 | | (0.93-1.20) | | 0.88 | |
| $20,000-$49,999 | 0.92 | (0.81-1.05) | | | 0.89 | | (0.75-1.05) | | 0.97 | | (0.87-1.07) | | 1.01 | | (0.89-1.15) | |  |  |
| $0-$19,999 | 0.92 | (0.77-1.10) | | | 0.84 | | (0.65-1.09) | | 0.97 | | (0.84-1.12) | | 1.06 | | (0.89-1.27) | |  |  |
| **Anger at Exam 1** |  |  | | |  | |  | |  | |  | |  | |  | |  | |
| Low | 1.07 | (0.96-1.19) | | | 1.09 | | (0.95-1.25) | | 1.04 | | (0.94-1.15) | | 1.14 | | (1.01-1.29) | | 0.55 | |
| Medium | 1.03 | (0.92-1.15) | | | 0.97 | | (0.83-1.15) | | 0.96 | | (0.87-1.07) | | 1.02 | | (0.88-1.17) | |  |  |
| High | 0.94 | (0.82-1.06) | | | 0.97 | | (0.83-1.12) | | 0.96 | | (0.86-1.08) | | 0.95 | | (0.83-1.10) | |  |  |
| **Depressive symptoms at Exam 1** |  |  | | |  | |  | |  | |  | |  | |  | |  | |
| No | 1.03 | (0.95-0.11) | | | 1.02 | | (0.93-1.12) | | 0.98 | | (0.91-1.04) | | 1.05 | | (0.96-1.14) | | 0.16 | |
| Yes | 0.91 | (0.74-1.12) | | | 0.83 | | (0.60-1.16) | | 1.10 | | (0.93-1.30) | | 0.94 | | (0.74-1.18) | |  |  |
| **Chronic stress at Exam 1** | | |  | | |  | |  | |  | |  | |  | |  | |  |
| Low | 1.07 | (0.97-1.18) | | | 1.03 | | (0.91-1.16) | | 1.02 | | (0.94-1.11) | | 1.13 | | (1.02-1.26) | | 0.22 | |
| Medium | 0.97 | (0.86-1.09) | | | 1.00 | | (0.85-1.19) | | 0.96 | | (0.86-1.07) | | 0.99 | | (0.85-1.14) | |  |  |
| High | 0.98 | (0.82-1.16) | | | 0.98 | | (0.76-1.26) | | 0.95 | | (0.81-1.12) | | 0.87 | | (0.71-1.06) | |  |  |
| **Discrimination at Exam 1** | | |  | | |  | |  | |  | |  | |  | |  | |  |
| Low | 0.97 | (0.87-1.08) | | | 0.91 | | (0.80-1.04) | | 1.01 | | (0.92-1.10) | | 1.02 | | (0.91-1.15) | | 0.74 | |
| Medium | 0.99 | (0.88-1.10) | | | 0.97 | | (0.84-1.13) | | 0.95 | | (0.86-1.05) | | 1.02 | | (0.90-1.17) | |  |  |
| High | 1.14 | (1.00-1.29) | | | 1.21 | | (1.03-1.43) | | 1.01 | | (0.89-1.14) | | 1.06 | | (0.91-1.24) | |  |  |
| **Neighborhood deprivation at Exam 1** | | | | | |  | |  | |  | |  | |  | |  | |  |
| Low | 0.92 | (0.78-1.09) | | | 0.87 | | (0.68-1.10) | | 1.00 | | (0.88-1.13) | | 0.95 | | (0.80-1.14) | | 0.71 | |
| Medium | 1.03 | (0.92-1.16) | | | 0.97 | | (0.83-1.13) | | 0.98 | | (0.88-1.09) | | 0.99 | | (0.89-1.11) | |  |  |
| High | 1.07 | (0.97-1.18) | | | 1.09 | | (0.96-1.23) | | 1.01 | | (0.93-1.11) | | 1.11 | | (0.97-1.27) | |  |  |
| **Neighborhood safety at Exam 1** | | |  | | |  | |  | |  | |  | |  | |  | |  |
| Safe | 1.02 | (0.94-1.10) | | | 1.00 | | (0.91-1.10) | | 1.00 | | (0.94-1.07) | | 1.02 | | (0.93-1.12) | | 0.11 | |
| Not safe | 1.10 | (0.93-1.30) | | | 0.92 | | (0.68-1.24) | | 0.93 | | (0.78-1.10) | | 1.10 | | (0.91-1.32) | |  |  |

Note: Each modified Poisson regression model accounted for clustering within neighborhood (i.e., census tract at Exam 1).

Assessment of effect measure modification of adjusted prevalence ratios* (aPR) for each visit for ideal or intermediate (no poor) metrics using the four biological Life’s Simple 7 metrics (BMI, blood pressure, cholesterol, and glucose) by levels of psychosocial risk measures and neighborhood social cohesion among participants included in the secondary analysis sample (N =7,291).

^*^ Adjusted for visit, age, sex/gender, race, nativity, geographic region, marital status, self-rated health, insurance, self-history of CVD and stroke, family history of CVD and stroke, social support, education, income, employment, anger, depressive symptoms, chronic stress, discrimination, neighborhood deprivation, neighborhood safety, and all possible product terms between visit, neighborhood social cohesion, and psychosocial risk

^†^ P-values were obtained from a global chi-squared test.

**Supplemental Table 8.** Assessment of effect measure modification of adjusted prevalence ratios* (aPR) using four biological Life’s Simple 7 metrics (secondary analysis sample)

| **Psychosocial risk measures**  **(Potential effect measure modifier)** | **High versus low neighborhood social cohesion and lower cardiovascular risk (0-1 poor metrics) by visit and level of psychosocial risk measure** | | | | | | | | **Medium versus low neighborhood social cohesion and lower cardiovascular risk (0-1 poor metrics) by visit and level of psychosocial risk measure** | | | | | | | | **p**^†^ | |
| --- | --- | --- | --- | --- | --- | --- | --- | --- | --- | --- | --- | --- | --- | --- | --- | --- | --- | --- |
|  | **Visit 1** | | | **Visit 2** | | | | | **Visit 1** | | | | **Visit 2** | | | |  | |
|  | **aPR** | **95% CI** | | | **aPR** | | **95% CI** | | **aPR** | | **95% CI** | | **aPR** | | **95% CI** | |  | |
| **Education at Exam 1** |  |  | | |  | |  | |  | |  | |  | |  | |  | |
| College degree or more | 1.01 | (0.97-1.05) | | | 1.01 | | (0.97-1.06) | | 1.01 | | (0.97-1.05) | | 0.99 | | (0.95-1.04) | | 0.96 | |
| High school or some college | 0.99 | (0.95-1.04) | | | 1.00 | | (0.94-1.06) | | 1.02 | | (0.98-1.06) | | 1.00 | | (0.95-1.05) | |  |  |
| Less than high school | 0.94 | (0.85-1.04) | | | 0.91 | | (0.80-1.02) | | 1.03 | | (0.96-1.11) | | 0.98 | | (0.90-1.07) | |  |  |
| **Employment at Exam 1** |  |  | | |  | |  | |  | |  | |  | |  | |  | |
| Employed | 1.01 | (0.97-1.05) | | | 1.01 | | (0.97-1.06) | | 1.02 | | (0.98-1.06) | | 1.01 | | (0.97-1.05) | | 0.69 | |
| Unemployed | 0.98 | (0.94-1.02) | | | 0.97 | | (0.91-1.02) | | 1.01 | | (0.98-1.05) | | 0.97 | | (0.93-1.01) | |  |  |
| **Income at Exam 1** |  |  | | |  | |  | |  | |  | |  | |  | |  | |
| $50,000+ | 1.01 | (0.97-1.05) | | | 1.03 | | (0.99-1.08) | | 1.02 | | (0.98-1.06) | | 0.99 | | (0.95-1.03) | | 0.07 | |
| $20,000-$49,999 | 1.02 | (0.97-1.08) | | | 0.96 | | (0.90-1.03) | | 1.04 | | (0.99-1.09) | | 0.98 | | (0.93-1.04) | |  |  |
| $0-$19,999 | 0.91 | (0.84-0.99) | | | 0.92 | | (0.83-1.02) | | 0.98 | | (0.93-1.03) | | 1.03 | | (0.96-1.10) | |  |  |
| **Anger at Exam 1** |  |  | | |  | |  | |  | |  | |  | |  | |  | |
| Low | 1.00 | (0.96-1.05) | | | 1.02 | | (0.96-1.08) | | 1.02 | | (0.97-1.06) | | 1.01 | | (0.96-1.06) | | 0.45 | |
| Medium | 0.99 | (0.95-1.04) | | | 0.99 | | (0.94-1.04) | | 1.01 | | (0.97-1.05) | | 0.96 | | (0.91-1.00) | |  |  |
| High | 0.98 | (0.93-1.04) | | | 0.95 | | (0.88-1.02) | | 1.02 | | (0.97-1.07) | | 1.01 | | (0.96-1.07) | |  |  |
| **Depressive symptoms at Exam 1** |  |  | | |  | |  | |  | |  | |  | |  | |  | |
| No | 0.99 | (0.96-1.03) | | | 1.00 | | (0.97-1.04) | | 1.01 | | (0.98-1.04) | | 0.99 | | (0.96-1.02) | | 0.27 | |
| Yes | 1.01 | (0.92-1.11) | | | 0.89 | | (0.78-1.03) | | 1.05 | | (0.97-1.13) | | 1.01 | | (0.92-1.11) | |  |  |
| **Chronic stress at Exam 1** | | |  | | |  | |  | |  | |  | |  | |  | |  |
| Low | 1.02 | (0.98-1.07) | | | 1.00 | | (0.96-1.04) | | 1.03 | | (0.99-1.06) | | 0.99 | | (0.95-1.02) | | 0.64 | |
| Medium | 0.97 | (0.92-1.02) | | | 1.00 | | (0.94-1.07) | | 1.02 | | (0.97-1.07) | | 1.00 | | (0.95-1.06) | |  |  |
| High | 0.96 | (0.90-1.03) | | | 0.95 | | (0.87-1.04) | | 0.99 | | (0.93-1.05) | | 0.99 | | (0.91-1.07) | |  |  |
| **Discrimination at Exam 1** | | |  | | |  | |  | |  | |  | |  | |  | |  |
| Low | 0.98 | (0.94-1.02) | | | 0.99 | | (0.94-1.03) | | 1.00 | | (0.97-1.05) | | 0.97 | | (0.92-1.01) | | 0.36 | |
| Medium | 0.99 | (0.95-1.04) | | | 1.00 | | (0.95-1.06) | | 1.01 | | (0.97-1.06) | | 0.98 | | (0.93-1.03) | |  |  |
| High | 1.02 | (0.96-1.07) | | | 0.99 | | (0.92-1.06) | | 1.03 | | (0.98-1.08) | | 1.04 | | (0.97-1.11) | |  |  |
| **Neighborhood deprivation at Exam 1** | | | | | |  | |  | |  | |  | |  | |  | |  |
| Low | 0.98 | (0.92-1.04) | | | 0.98 | | (0.90-1.07) | | 0.98 | | (0.93-1.04) | | 0.94 | | (0.87-1.02) | | 0.77 | |
| Medium | 1.00 | (0.94-1.06) | | | 0.97 | | (0.92-1.03) | | 1.02 | | (0.97-1.07) | | 1.00 | | (0.96-1.06) | |  |  |
| High | 1.01 | (0.97-1.05) | | | 1.03 | | (0.98-1.07) | | 1.03 | | (1.00-1.07) | | 1.01 | | (0.97-1.05) | |  |  |
| **Neighborhood safety at Exam 1** | | |  | | |  | |  | |  | |  | |  | |  | |  |
| Safe | 1.00 | (0.97-1.04) | | | 1.01 | | (0.97-1.04) | | 1.02 | | (0.99-1.05) | | 1.00 | | (0.96-1.03) | | 0.57 | |
| Not safe | 0.96 | (0.88-1.04) | | | 0.89 | | (0.78-1.02) | | 1.01 | | (0.95-1.07) | | 0.96 | | (0.89-1.04) | |  |  |

Note: Each modified Poisson regression model accounted for clustering within neighborhood (i.e., census tract at Exam 1).

Assessment of effect measure modification of adjusted prevalence ratios* (aPR) for each visit for lower cardiovascular risk (0-1 poor metrics) compared with high cardiovascular risk (2-4 poor metrics) using the four biological Life’s Simple 7 metrics (BMI, blood pressure, cholesterol, and glucose) by levels of psychosocial risk measures and social support among MESA and JHS participants included in the secondary analysis sample (N =7,243).

^*^ Adjusted for visit, age, sex/gender, race, nativity, geographic region, marital status, self-rated health, insurance, self-history of CVD and stroke, family history of CVD and stroke, education, income, employment, anger, depressive symptoms, chronic stress, discrimination, neighborhood deprivation, neighborhood safety, and all possible product terms between visit, social support, and psychosocial risk.

^†^ P-values were obtained from a global chi-squared test
